# Improving the Stability of Halide Perovskite Solar Cells Using Nanoparticles of Tungsten Disulfide

**DOI:** 10.3390/nano12244454

**Published:** 2022-12-15

**Authors:** Philip Nathaniel Immanuel, Song-Jeng Huang, Viktor Danchuk, Anastasiya Sedova, Johnathan Prilusky, Achiad Goldreich, Hila Shalom, Albina Musin, Lena Yadgarov

**Affiliations:** 1 Department of Mechanical Engineering, National Taiwan University of Science and Technology, Taipei 106, Taiwan; 2 Department of Chemical Engineering, Faculty of Engineering, Ariel University, Ariel 4076414, Israel; 3 Physics Department, Faculty of Natural Sciences, Ariel University, Ariel 4076414, Israel

**Keywords:** methylammonium lead iodide, transition metal dichalcogenides, solar cells, halide perovskite, renewable energy

## Abstract

Halide perovskites-based solar cells are drawing significant attention due to their high efficiency, versatility, and affordable processing. Hence, halide perovskite solar cells have great potential to be commercialized. However, the halide perovskites (HPs) are not stable in an ambient environment. Thus, the instability of the perovskite is an essential issue that needs to be addressed to allow its rapid commercialization. In this work, WS_2_ nanoparticles (NPs) are successfully implemented on methylammonium lead iodide (MAPbI_3_) based halide perovskite solar cells. The main role of the WS_2_ NPs in the halide perovskite solar cells is as stabilizing agent. Here the WS_2_ NPs act as heat dissipater and charge transfer channels, thus allowing an effective charge separation. The electron extraction by the WS_2_ NPs from the adjacent MAPbI_3_ is efficient and results in a higher current density. In addition, the structural analysis of the MAPbI_3_ films indicates that the WS_2_ NPs act as nucleation sites, thus promoting the formation of larger grains of MAPbI_3_. Remarkably, the absorption and shelf life of the MAPbI_3_ layers have increased by 1.7 and 4.5-fold, respectively. Our results demonstrate a significant improvement in stability and solar cell characteristics. This paves the way for the long-term stabilization of HPs solar cells by the implementation of WS_2_ NPs.

## 1. Introduction

The ongoing energy crisis, combined with global warming and air pollution, indicates the urgent need to develop cost-effective, environmentally stable and green energy harvest technologies. Solar cells present the most promising paths for green energy harvest in this context. Photovoltaic solar cells are already a part of our life, especially in countries where solar energy is available most of the year. Solar cells make low-cost electricity available for the world’s population, particularly for third-world countries. Recently silicon-based solar cells have overtaken the market, but the search for new and more efficient materials is ongoing.

In recent years halide perovskite solar cells (PSCs) have been the center of attention [1,2,3], reaching an efficiency of 25.7% [4]. This high efficiency and low cost make PSCs great candidates for entering the photovoltaic solar cell market [5]. Halide perovskites (HPs) are very promising due to their high absorption coefficient in the visible region [6], low exciton binding energy [7] and long electron-hole diffusion lengths [8]. The general formula of HPs is ABX_3_, where A is usually cesium (Cs) or methylammonium (MA), B is Pb or Sn, and X = I, Br, or Cl [9] The most commonly studied are methylammonium lead trihalide (CH_3_NH_3_PbX_3_) perovskite with an optical bandgap between 1.5 and 2.3 eV depending on halide type [10,11].

Generally, PSCs are composed of thin layers with different functions for accomplishing four basic processes: (1) absorption of photons with a suitable material, (2) creation of free charge carriers: separation of electron-hole pair by internal field, (3) collection and (4) transportation of photo-generated charge carriers through an electrical circuit (Scheme S1, SI). The n-i-p PSCs architecture commonly involves a planar heterojunction [12] with compact titanium oxide (TiO_2_) or tin oxide (SnO_2_) as electron transport layers (ETL) [9]. The ETL and the hole transport layer (HTL) assist in separating the electron-hole pairs [1] generated in the perovskite absorber [13].

There are still challenges in fabricating high-performance and stable PSCs for market-oriented fabrication. The principal challenges of PSCs are improving absorption and overcoming the instability caused by moisture, oxygen and heat. One of the possible solutions is adding a transition metal dichalcogenide (TMD) material to improve absorption and stability. TMDs have outstanding stability, ability to conduct both holes and electrons, and high absorption coefficient [14,15,16].

These two-dimensional (2D) materials consist of an S–M–S sandwich-like structure, where the transition metal atom (M = W, Mo) are bonded to the chalcogen (S = S, Se, or Te) atoms via strong covalent bonds. The MS_2_ layers are stacked together by weak van der Waals forces [17,18] The indirect bandgap of the bulk and multilayer MS_2_ converts into a direct one in a single layer [19,20]. Due to a coupling between their mechanical and electrical properties, the semiconducting characteristics of 2D-TMDs are engineered by inducing mechanical, structural, or morphological changes [21,22,23]. The MoS_2_ flakes implemented in the PSCs act as a buffer layer and lead to stable devices. The presence of the MoS_2_ suppresses the recombination of electron holes at the HTL/perovskite and ETL/perovskite transport layer interfaces, assists in the charge transfer, and prevents the degradation of the perovskite layer due to the formation of the larger grains [24,25]. In the general PSC scheme, the MoS_2_ flakes function mainly as stabilizers and are also used for the transportation of photo-generated charge carriers (fourth basic process). Nevertheless, the exfoliation of MoS_2_ flakes from the bulk material is time-consuming and might last up to several days [24,25,26].

Closed-cage TMDs nanostructures sustain all the properties of the bulk but are much more stable [27,28] due to the lack of dangling bonds and thus can suppress exciton capture at non-radiative recombination centers, such as surface states and impurities. Due to their sizable bandgaps, strongly bound excitons and high oscillator strength, these materials introduce strong light-matter interaction (polaritons) under ambient conditions [27,29,30,31,32] The strong light-matter interaction of WS_2_/MoS_2_ may further boost the PSCs performance as the plasmonic and polaritonic nanomaterials are often used to boost the efficiency and absorption of the active layers [33]. The polaritons can enhance light absorption, either by electromagnetic field enhancement near the particle or by light scattering into trapped optical modes [34]. In this work, the WS_2_ NPs function in the absorption and transportation of photo-generated charges as well. (first and fourth basic processes).

Here, we compare the properties of the absorption layer of the fabricated PSCs with and without the addition of n-type WS_2_ NPs, [35,36] and MAPbI_3_. By introducing the WS_2_ NPs between the ETL and the active perovskite layer, we obtain a 4.5-fold improvement in the stability of the MAPbI_3_-absorbing layer. Moreover, we observe increased absorption and improved PSCs characteristics upon adding 1 wt% of the WS_2_ NPs. The beneficial impact can be explained by the stability and the high absorption coefficient of the WS_2_ NP. Another advantage is the overlap between the energy bands, where the upper energy levels of the WS_2_ NPs and MAPbI_3_ match. This overlap may promote a charge transfer from MAPbI_3_ to WS_2_, thus improving charge separation. Moreover, the increase in the absorption of the WS_2_ NPs/MAPbI_3_ layers contributes to the generation of electron-hole pairs. We also observe that the WS_2_ NPs act as nucleation sites, resulting in larger MAPbI_3_ grains, thus hindering nonradiative energy losses. With this study, our results pave the way toward the stabilization of MAPbI_3_-based PSCs.

## 2. Materials and Methods

### 2.1. Materials

FTO-covered glass (6 Ω/cm^2^) was purchased from Biotain Hong Kong Co. Limited. Isopropanol (99%) and bi-distilled water (BDW, Milli-Q^®^ IQ 7003) were purchased from Merk, Darmstadt, Germany. Ethanol (99.8%) was purchased from Acros Organics. Chlorobenzene and CH_3_NH_3_ were purchased from Sigma-Aldrich, USA. Spiro-MeOTAD was purchased from Ossila, Sheffield, UK. Ti-Nanoxode BL/SC was purchased from Solaronix S. A., Switzerland. Ag wire 99.99% was purchased from Advent, UK, PbI_3_ (Alfa Aesar, Massachusetts, UK) and used without further purification.

### 2.2. Solar Cell Fabrication

#### 2.2.1. Fabrication of WS_2_ NPs

The multiwall WS_2_ NPs are synthesized according to the procedure reported by Tenne et al. [37,38] In detail, on a quartz boat, WO_3_ nano-powders (100 nm in dimension) were spread evenly and maintained in a 1%H_2_/99%N_2_ gas environment at 900 °C. The WO_3_ was reduced to WO_3−x_ (intermediate phases) by the H_2_ gas. Following this reaction, the WO_3−x_ was sulfurized by 1%H_2_/99%N_2_ and H_2_S gas to obtain the fully formed WS_2_ NPs.

#### 2.2.2. Fabrication of Solar Cells

For solar cell fabrication, the FTO-covered glass substrates with size 2.5 cm × 2.5 cm × 2.2 mm are first etched in 3M hydrochloric acid using a special mask of the desired geometry for the electrodes. The etched substrates are then cleaned using an ultrasonic bath (GT Sonic-P3, Guangdong, China) in a cleaning solution (Micro-90 International Products Corporation, USA, diluted with distilled water, 1:50) water-soap solution (2%), distilled water, isopropanol, and deionized water (BDW) for 10 min at 80 °C in each solvent. After each sonication cycle, the substrates are dried with dry air. Then the FTO glass substrates are immersed three times in boiled BDW DI water and dried with nitrogen (N_2_). Finally, the FTO substrates are thermally treated at 120 °C for 20 min in a Vacuum Drying Oven DZ-1BC11 (Faithful Inst. Co., China) under 5 × 10^−21^ Torr pressure. Clean substrates were stored in a desiccator under a vacuum. Before the ETL deposition, the substrates were transferred to a UV-Ozone cleaner for 15–30 min. To cover the FTO substrate with ETL, we drop cast 80 μL Ti-Nanoxode BL/SC (Solaronix, S. A., Switzerland) (TiO_2_, blocking layer) in a static regime, followed by a 30 s spin coating at the speed of 5000 rpm and acceleration of 2500 cycles/s^2^ in the spin coater (WS-650HZ-23NPPB, Laurell Technologies Inc, Lansdale, PA, USA). The FTO/TiO_2_ substrates are then annealed at 550 °C for one hour under continuous dry airflow (600 sccm) in the Tube Furnace KJ-T1200 (Kejia Furnace Co., Ltd., Henan, China).

After 30 s of plasma treatment EQ-PCE3 (MTI Corp., USA) at a “High” regime in residual air at 0.18 torr., 200 μL of WS_2_ NPs/ethanol solution (0.07 g/L) was deposited on the FTO/TiO_2_ substrate by spin-coating at the speed of 3000 rpm and then thermally treated in vacuum at 120 °C for 15 min. In the next step, 60 μL MAPbI_3_ perovskite precursor solution was applied by spin coating under a controlled N_2_ environment in a nitrogen-filled glove box VTI (Vacuum Technology Inc., Oak Ridge, USA) on the formed FTO/TiO_2_/WS_2_ NPs films in an antisolvent strategy [39], modified for double antisolvent treatment. The dots A and B mark the timing of the chlorobenzene (antisolvent) pouring on the top of the forming MAPbI_3_ layer, correspondingly 500 and 250 μL. This process produces films of MAPbI_3_ with thickness of layer ranging between 300 and 500 nm.

After annealing at 90 °C for 10 min, and cooling to 28 °C, 60 μL of Spiro-MeOTAD was spin-coated at 4000 rpm for 30 sec on the FTO/TiO_2_/WS_2_ NPs/MAPbI_3_ in the dynamic regime. That process produces films of Spiro-MeOTAD with thickness of layer ranging between 300 and 400 nm (Figure S1). In the final step, Ag contact electrodes were deposited by vacuum thermo-resistive evaporation JEE—4B (JEOL, Tokyo, Japan). To avoid Ag atoms’ penetration through the HTL layer to the MAPbI_3_ causing cell damage, the initial 10 nm of Ag electrodes were deposited at a rate of 1 nm/min and the remaining 110 nm at a rate of 55 nm/min.

### 2.3. Characterization

X-ray Diffraction (XRD) patterns are measured using Rigaku SmartLab diffractometer, Tokyo, Japan. The diffractometer is equipped with a Cu anode (CuKα radiation with 1.5418 Å wavelength, 10–80° 2θ range, step width 0.01°) operated at 40 kV, 120 mA. The scans are acquired in 2-Theta Bragg-Brentano mode. The perovskite films’ thickness and surface morphology were revealed with scanning electron microscopy (SEM) MAIA3 (TESCAN, Brno, Czech Republic). The scattering spectra are acquired with Jasco V-750 spectrophotometer in the range of 420 nm to 800 nm. These measurements are used for the band gap calculations. We use a modified Tauc equation to extract the band gap values [40,41].

The PSCs are characterized by a solar simulator (Abet Sun 2000, class A) under standard conditions AM1.5G. The measurements are carried out with an assistance of a Keithley 2400 Sourcemeter (Cleveland, Ohio, United States) and a custom-made program LabView-based. The power conversion efficiency (PCE), short-circuit current density (J_cs_), open-circuit voltage (V_OC_) and the fill factor (FF) are calculated from the current density-voltage characteristics (J-V).

### 2.4. Simulation

**Scheme 1:**
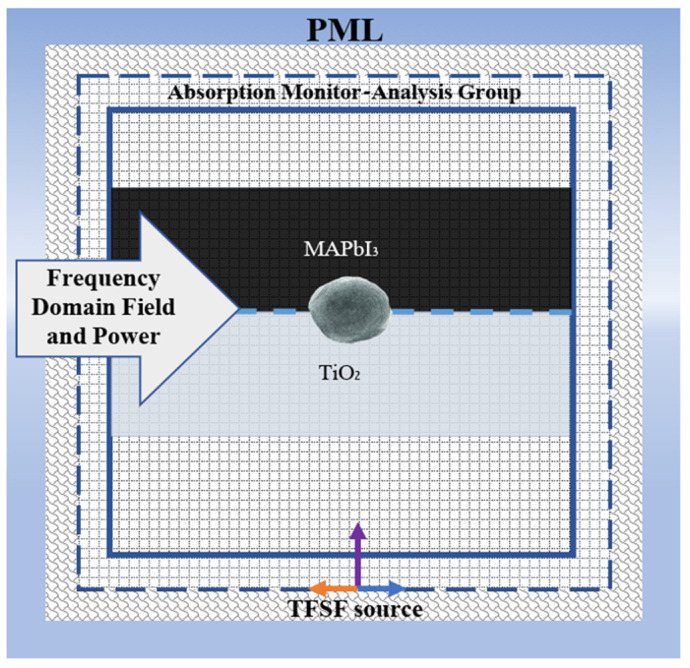
Cross section view of the simulation system.

Commercial software finite-difference time-domain method (FDTD) (Ansys Lumerical FDTD) was used to perform the simulations [42]. Here, Maxwell’s equations are solved in time and space with various mesh sizes. A broadband Total-Field Scattered-Field (TFSF) source is used in the wavelength domain (400–800 nm) to measure the local electric field. The electric field response from the materials is captured using a 2D monitor. The frequency domain field and power monitor directly measure the local field enhancement. The cross-section of the simulation scheme is presented in Scheme 1.

The embedded WS_2_ NP at the interface of TiO_2_ and MAPbI_3_ is modeled as a sphere inside a dielectric material. The model, without the WS_2_ NP, is considered the reference model and the signal is considered as the reference signal. We examine the size effect of the WS_2_ nanoparticle (10–80 nm). The background index of the FDTD region was set to be n = 1 for air. The dielectric functions of MAPbI_3_, TiO_2_, and WS_2_ are extracted from [43,44,45] In this simulation system, the mesh order of the NP was set to 2, and the TiO_2_ and MAPbI_3_ mesh orders were 3 [46].

### 2.5. Stability Measurements

We studied the stability of the MAPbI_3_ layers with and without WS_2_ NPs by exposing the sample to light and ambient environment. The WS_2_ NP and MAPbI_3_ thin layers were produced using the procedure described for solar cell in Section 2.2.2. Several stability tests were performed. (1) The MAPbI_3_ layers on top of the FTO glass with and without WS_2_ NPs were exposed to air for one hour and sealed and stored in a vacuum standard vacuum drawer for one month. (2) Here, the examined materials were placed on the glass, not on FTO. All the samples were vacuum sealed in a petri dish and kept under sunlight for 11 days. (3) The samples were vacuum sealed in a plastic bag and exposed to sunlight. The reflection of all the samples from test 2 and 3 was measured every few days and the samples were re-sealed under vacuum. For test 1, the samples were photographed after one month. To analyze the results, the reflection of samples from tests 2 and 3 were converted to absorption using the following formula A = 1−R, where A stand for the absorption and R denotes the reflection. The measured spectra are presented in Figures S2a,b and S3a–c. For all the measured spectra the intensity at 700 nm was chosen for the absorption intensity comparison of the samples.

Additionally, the performance of the PSCs was examined in two other configurations by placing the WS_2_ NPs (1) into the MAPbI_3_ precursor and (2) in between the HTL and MAPbI_3_. Here, the results were very inconclusive and thus not presented in this work.

## 3. Results and Discussion

**Figure 1:**
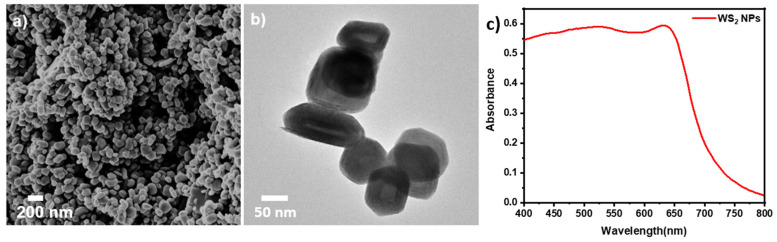
(**a**) SEM image of WS_2_ NPs, (**b**) TEM image of WS_2_ NPs. (**c**) absorption spectrum of WS_2_ NPs.

The morphology of the WS_2_ NPs was studied by SEM and TEM, as shown in Figure 1a,b. The average dimension of the NPs is in the range of ~60 to 120 nm in diameter. [28] The closed cage WS_2_ hollow nanoparticles appear to be crystalline and faceted. The absorption spectrum of the WS_2_ NPs has two peaks at 630 and 530 nm, as shown in Figure 1c. These two peaks are due to the two excitonic transitions in WS_2_ NPs. [28]

**Figure 2:**
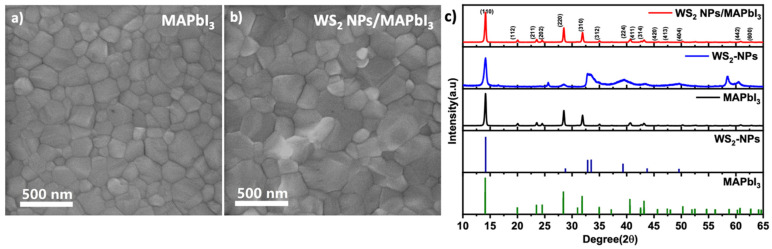
(**a**) SEM image of MAPbI_3_, (**b**) SEM image of WS_2_ NPs/MAPbI_3_, and (**c**) XRD patters of the MAPbI_3_, WS_2_ NPs, and WS_2_ NPs/MAPbI_3_.

To investigate the influence of WS_2_ NPs on MAPbI_3_ films, the morphological changes, and the crystalline phase of the films was studied by SEM and XRD, respectively. The SEM images of MAPbI_3_ and WS_2_ NPs/MAPbI_3_ films are shown in Figure 2a,b. The average grain size of the pristine MAPbI_3_ layer is smaller (247.48 ± 56.28 nm) and narrow in distribution (Figure S4a). However, the MAPbI_3_ deposited on top of the WS_2_ NPs has large grains and wide distribution (271.17 ± 55.44 nm) (Figure S4b). These results indicate that adding WS_2_ NPs increases the MAPbI_3_ grain size. Moreover, the absorbance of the HPs is enhanced due to the high absorption coefficient of the WS_2_ NPs (1 × 10^6^ cm^−1^) [47]. The WS_2_ NPs contributes to increased absorption, as well as the enlarged grain size, which leads to enhanced photocurrent [48]. The absorption of photons causes electrons(e-) to eject and produce photocurrent. When the absorbance is enhanced, more photons will be absorbed and more electrons will be ejected, which results in a high photocurrent. Thus, the absorption enhances the photocurrent. [48] The XRD results (Figure 2c) show that the formed MAPbI_3_ film with and without the WS_2_ NPs contains ~99.5% of the tetragonal MAPbI_3_ phase [49,50] The close analysis of the diffraction patterns reveals only a minute amount (~0.5%) of the monoclinic lead iodide phase. That phase is present in the thin MAPbI_3_ layer due to the incomplete conversion to the perovskite phase [51].

To understand the effect on grain size and crystalline phases and to find the optimal concentration of WS_2_ NPs, the MAPbI_3_ films were prepared with different weight percentages (wt%) of WS_2_ NPs (1%, 2%, and 3%). The morphology and the phase were examined by SEM and XRD (Figures S5a–c and S6), respectively. Interestingly, the XRD spectra show no indication of WS_2_ NPs presence in the MAPbI_3_ films, even upon increasing the WS_2_ NPs concentration (Figure S6). This can be assigned to the overlap between the (110) and (002) peaks of MAPbI_3_ and WS_2_ NPs, respectively. Due to the low WS_2_ NPs concentration, the lower intensity peaks of WS_2_ are hidden in the MAPbI_3_ spectral background. According to SEM analysis, there is no significant difference in the grain size (268.58 ± 63.20) between the 1 wt% and when the WS_2_ NPs concentration was double (2 wt%) (Figure S5d). When the concentration tripled, the average grain size became smaller (200.41 ± 69.6 nm). The changes in the grain size can be explained by the aggregation of the NPs. Namely, when the concentration of the WS_2_ NPs was higher than 1 wt%, more NPs aggregate form and act as multiple nucleation centers. The increase in the number of the formed aggregates is further verified by SEM analysis of the WS_2_ NPs deposited on a substrate with different concentrations (1 and 3 wt%) (Figure S7a,b). The combination of SEM and XRD analysis indicates that adding 1 wt% of WS_2_ NPs to the MAPbI_3_ films produces larger grains of the latter without changing its phase. The larger grain size has a beneficial impact on the absorption, stability and efficiency of the PSCs [25,52,53]. Moreover, it reduces the number of grain boundaries and thus decreases the non-radiative processes [48], and enhances the absorption and photocurrent [48]. Hence, for further experiments and characterization, we use 1 wt% of WS_2_ NPs to prepare MAPbI_3_ films and PSCs.

**Figure 3:**
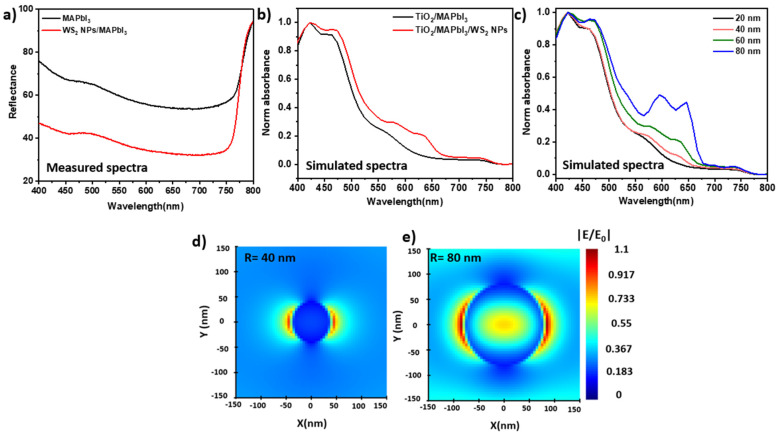
(**a**) Reflectance spectrum of MAPbI_3_ and WS_2_ NPs/MAPbI_3_, (**b**) simulated absorption spectrum of MAPbI_3_/WS_2_ NPs and MAPbI_3_/WS_2_ NPs/TiO_2_, (**c**) simulated absorption spectrum of WS_2_ NPs with different radius, (**d**,**e**) simulated electric field distribution at 710 nm of WS_2_ NPs with radius of (**d**) 40 nm (**e**) 80 nm.

To study the optical properties of the hybrid system, the reflectance of the pristine MAPbI_3_ films and WS_2_ NPs/MAPbI_3_ were examined. Impressively, the measured reflectance shows a 1.7-fold reduction in the reflectance upon the addition of 1 wt% of WS_2_ NPs. (Figure 3a) These results indicate that the WS_2_ NPs/MAPbI_3_ layers absorb much more photons compared to the pristine MAPbI_3_. Importantly, the low content of the added WS_2_ NPs increases the absorbance but does not affect the band gap of the MAPbI_3_ (Figure S8). Here, the band gap of both hybrid and pristine films is 1.63 eV, which is characteristic of MAPbI_3_ [10].

To understand the impact of particle size and concentration of the WS_2_ NPs on absorbance, we simulate the absorption spectra of the MAPbI_3_ and WS_2_ NPs/MAPbI_3_ on TiO_2_ substrate (120 nm). The simulations were performed using commercial software: Ansys Lumerical FDTD [54,55,56]. The TiO_2_ substrate was chosen to consider the relevant contribution of the relevant interfaces, namely the ETL and the absorbing layer (MAPbI_3_). The absorption spectra of MAPbI_3_ on TiO_2_ with and without WS_2_ NPs are presented in Figure 3b. The additional peaks at 550 nm to 720 nm (red line) are due to the contribution of WS_2_ NPs to the MAPbI_3_ absorbance. The simulation results further support the experimental observations. Here again, the absorption of the MAPbI_3_ film increases upon the addition of the WS_2_ NPs. Nevertheless, a substantial difference between the experimental and the simulated spectra are clearly observed. One of the reasons is the substrate. Namely, the measured film is placed on the glass, where the simulated film is located on TiO_2_. The experimental measurement was on a glass substrate, but the simulation was performed with the background of TiO_2_. An additional reason is a difference in the measurement setup: standard spectrophotometer vs. perfect simulation conditions. Notwithstanding, the simulation results manifest the beneficial impact of WS_2_ NPs on the increased absorbance of the MAPbI_3_ films.

Additionally, we simulated the absorption of the WS_2_ NPs with different radii to study the impact of dimension and concentration on the enhancement factor (Figure 3c–e). The absorption intensity increases with the radius of the WS_2_ NPs (Figure 3c). Furthermore, the electric field (|E/E_0_|^2^) of the larger WS_2_ NP (d = 80 nm) is much broader and more intense compared to the one of d = 40 nm. The absorption intensity and the |E/E_0_|^2^ fields are higher for the larger NPs, which is beneficial for PSCs performance.

**Figure 4:**
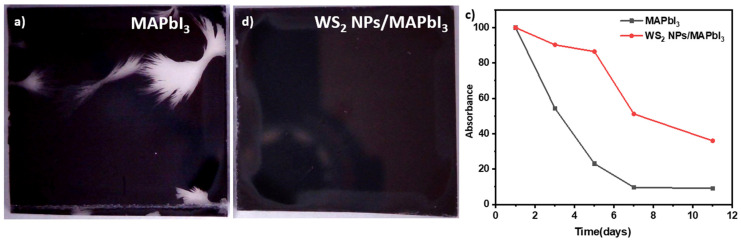
(**a**) MAPbI_3_ and (**b**) WS_2_ NPs/MAPbI_3_ layers on top of the FTO glass substrate after exposure to air, (**c**) stability measurement of the samples: the absorption percentage of the hybrid WS_2_ NPs/MAPbI_3_ (red line) and the pristine MAPbI_3_ (black line).

Next, we study the influence of WS_2_ NPs on the stability of MAPbI_3_ films by examining the latter in a vacuum and under sunlight. For the preliminary stability test, the MAPbI_3_ layers on FTO glass with and without the WS_2_ NPs were examined. The MAPbI_3_ layers were exposed to air for one hour, then sealed and stored in darkness for one month. The microscope images of the MAPbI_3_ and WS_2_ NPs/MAPbI_3_ are presented in Figure S9. Impressively, the MAPbI_3_ layers appear with cracks (Figure 4a), while the WS_2_ NPs/MAPbI_3_ layers remain undamaged (Figure 4b). These results demonstrate positive long-term stability effects of the WS_2_ NPs on the MAPbI_3_ films.

Following the successful preliminary results, the long-term stability of WS_2_ NPs/MAPbI_3_ and MAPbI_3_ films under sunlight in a vacuum was examined. The samples were placed in a petri dish, vacuum sealed and exposed to sunlight for eleven days. The absorption of the films was measured every few days (Figures S3a,b and S10). Figure 4c shows the comparison between the absorption percentage of the hybrid WS_2_ NPs/MAPbI_3_ and the pristine MAPbI_3_. The absorption of both films decreases upon exposure to sunlight. However, while the absorption of MAPbI_3_ film decreased by almost 50% in the first 3 days, the absorption of the hybrid film decreases only by 10%. This trend proceeds and the absorption of the MAPbI_3_ film decreases by 90% of the initial intensity in seven days. Outstandingly, the absorption of the WS_2_ NPs/MAPbI_3_ films remains high and starts to drop only after 5 days. Moreover, on the eleventh day, the absorption percentage of the hybrid was as high as 40% whereas the absorption of MAPbI_3_ film was only 10%. These excellent results indicate that a minute amount of WS_2_ NPs dramatically stabilizes the MAPbI_3_. Additionally, we examined the stability of the MAPbI_3_ and WS_2_ NPs/MAPbI_3_ film in a vacuum sealed bag under sunlight, and the results are presented in Figure S3a–c. Here again, the hybrid films exhibit better stability compared to the pristine ones (MAPbI_3_). The phenomenon’s proposed mechanism includes charge separation [57] and heat dissipation [58]. The WS_2_ thermal conductivity is high, so it dissipates heat rapidly. Furthermore, the charge separation occurred in the HPs by transferring the photoexcited charges to WS_2_ NPs [57,59]. The observed stabilization impact of a minute amount of WS_2_ on MAPbI_3_ films is extraordinary. Namely, 1 wt% of WS_2_ NPs is sufficient to stabilize the WS_2_ NPs/MAPbI_3_ films by 4.5-fold.

**Figure 5:**
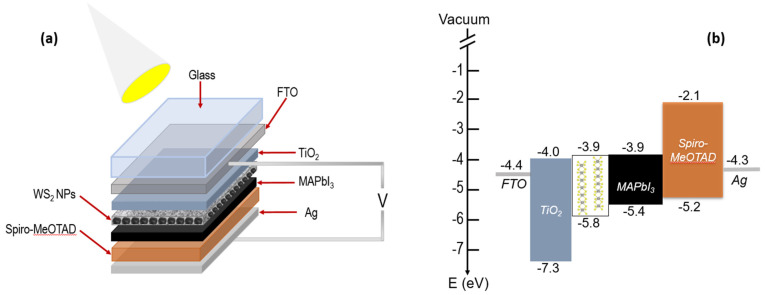
(**a**) configuration of WS_2_ NPs/MAPbI_3_ heterostructure-based PSCs, and (**b**) schematic energy level diagram.

Finally, the WS_2_/PSCs performance was examined using the J-V characteristics. The Figure 5a illustrate a schematic representation of the WS_2_ NPs/MAPbI_3_-based PSCs, where MAPbI_3_ is deposited on top of the WS_2_ NPs. In this configuration, the formed MAPbI_3_ layer is in contact with the n-typseparatio [35,36] and with TiO_2_ ETL. According to the energy levels diagram of the formed layers in Figure 5b, [23,60,61] the upper energy levels of the MAPbI_3_ and WS_2_ NPs are practically matching. This energetic match allows a fast electron transition from the MAPbI_3_ to the n-type WS_2_ NPs. [59] Moreover, the hole diffusion is most likely heading from WS_2_ NPs to the MAPbI_3_ and to the Spiro-OMeTAD polymer.

**Figure 6:**
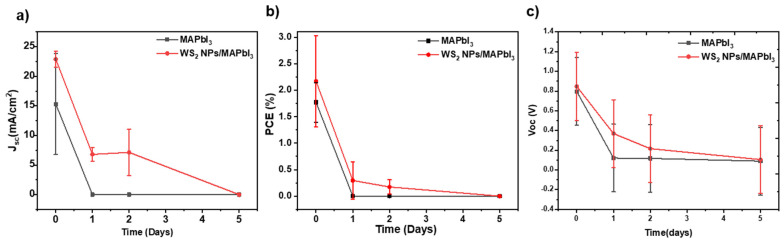
(**a**) J-V characteristic, (**b**) PCE and (**c**) V_oc_ of MAPbI_3_ and WS_2_ NPs/MAPbI_3_ PSCs for different days.

We analyze the performance of the PSCs with the absorbing layer of MAPbI_3_ and compare it with WS_2_ NPs/MAPbI_3_. Here the vastly studied MAPbI_3_-based PSC serves as a reference cell. The measured values are somewhat low compared to the results presented in the literature [25,62] This can be explained by using an unstable hole transport layer and non-optimal experimental conditions. In the future, the photovoltaic parameters of our devices can be boosted by replacing the Ag contacts with gold (Au) or platinum (Pt) [63]. The PSCs might also be improved by refining the conditions, such as using a more efficient and stable hole transport layer and electron transport layer. The characteristic performance for three PSCs was measured on selective days and the average values are presented in Figure 6. Moreover, compared to the reference, the hybrid samples exhibited higher values of the V_OC_, FF, J_sc,_ and, as a result, PCE (Table 1). The difference is 5.9% and 59.63% for the V_OC_ and PCE, respectively (Figure 6a–c).

Interestingly, we see that the J_SC_ tends to be higher for the cells with WS_2_ NPs/MAPbI_3_, supporting the idea of the efficient electron transfer from the MAPbI_3_ to WS_2_ NPs. Similar results are reported for the MoS_2_ buffer layer that assists in the transition of holes from MAPbI_3_ to Spiro-OMeTAD and the stabilization of the PSCs [24] One of the disadvantages of the MoS_2_ flakes implementation is the time-consuming process of their exfoliation from the bulk material. The time of this process ranges from 6 to 66 h, thus substantially increasing the time for fabrication of the PSCs devices [24,25,26]. Here, we solve this problem by using the WS_2_ NPs that can be incorporated as received. Moreover, the closed cage nanostructures are much more stable than the layered counterparts due to the fewer defects and lack of dangling bonds in the latter.

## 4. Conclusions

We demonstrate a conceptual study where we suggest using the WS_2_ NPs as an additive to the absorbing layer for improved stability and absorption of PSCs. Here, the WS_2_ NPs are used as a charge separation layer in the MAPbI_3_-based PSCs with compact TiO_2_. These NP_S_ can be incorporated as is and without any treatment, giving an advantage over the studies reported on MoS_2_ flakes to date. The results of our studies indicate that adding a minute amount of WS_2_ NPs increases the MAPbI_3_ grain size and enhances the overall absorbance, which leads to enhanced photocurrent. The simulation results show that the absorption is high for bigger NPs, which favors enhancing the absorption of the MAPbI_3_ layers. The high concentration of WS_2_ NPs leads to produce small grains due to aggregation, although the minute amount (1 wt%) of the WS_2_ NPs used for the PSCs devices produces larger grains and enhances the stability of MAPbI_3_ layers by 4.5-fold. These preliminary results show a clear potential for the use of WS_2_ NPs in the long-term stabilization of the PSCs.

## Figures and Tables

**Table 1:** Photovoltaic parameters of the devices without (MAPbI_3_) and with WS_2_ NPs (WS_2_ NPs/MAPbI_3_).

Device	V_OC_ (V)	J_SC_ (mA∙cm^−2^)	FF (%)	PCE (%)
MAPbI_3_	0.79 ± 0.3	15.30 ± 8.54	14.88	1.34 ±0.29
WS_2_ NPs/MAPbI_3_	0.84 ± 0.3	22.89 ±1.35	16.85	3.32 ± 0.61
Difference (%)	5.9%	33.1%	11%	59.63%

## Supplementary Materials

The following supporting information can be downloaded at: https://www.mdpi.com/article/10.3390/nano12244454/s1, Scheme S1. (a) absorption of photons with WS_2_ NPs/MAPbI_3_, (b) creation of free charge carriers: separation of electron-hole pair by internal field, (c) collection and (d) transportation of photo-generated charge carriers through an electrical circuit. Figure S1. Cross section SEM images of MAPbI_3_ layer. Figure S2. Stability measurement of (a) MAPbI_3_ and (b) WS_2_ NPs/MAPbI_3_ films in petri dish. Figure S3. Stability measurement of (a) MAPbI_3_, (b) WS_2_ NPs/MAPbI_3_ films on vacuum sealed bags, (c) absorption percentage of the hybrid WS_2_ NPs/MAPbI_3_ (red line) and the pristine MAPbI_3_ (black line). Figure S4. Grain size distribution of (a) MAPbI_3_ and (b) WS_2_ NPs/MAPbI_3_ with 1 wt%. Figure S5. (a–c) SEM images of the WS_2_ NPs/MAPbI_3_ layers with increased concentration of WS_2_ NPs. Here C1—1 wt% concentration of WS_2_ NPs, C2—2 wt% concentration of WS_2_ NPs, C3—3 wt% concentration of WS_2_ NPs. (d) Grain size distribution of (a) WS_2_ NPs/MAPbI_3_ for C2 concentration of NPs and (e) WS_2_ NPs/MAPbI_3_ for C3 concentration of NPs. (f) Absorbance spectrum of MAPbI_3_ with three grain size. Figure S6. XRD patterns of the WS_2_ NPs/MAPbI_3_ layers with increased concentration of WS_2_ NPs. Here C1—1 wt% concentration of WS_2_ NPs, C2—2 wt% concentration of WS_2_ NPs, C3—3 wt% concentration of WS_2_. Figure S7. (a) SEM images of WS_2_ NPs on glasses with C1 concentration and (b) SEM images of WS_2_ NPs on glasses with C3 concentration. Figure S8. Calculated band gap of MAPbI_3_ and WS_2_ NPs/MAPbI_3_. Figure S9. Light microscope images of three different magnifications of the (a–c) MAPbI_3_ and (d–f) WS_2_ NPs/MAPbI_3_ layers on the FTO glass after exposure to air. Figure S10. Picture of MAPbI_3_ films and WS_2_ NPs/MAPbI_3_ films after stability test under sun light.
