# Supplementary material for: Improving the Stability of Halide Perovskite Solar Cells Using Nanoparticles of Tungsten Disulfide
Source: Nanomaterials (Basel). 2022 Dec 15;12(24):4454. doi: 10.3390/nano12244454 (PMC9784750; doi:10.3390/nano12244454)
Supplement: Supplementary file 1 [file nanomaterials-12-04454-s001.zip › nanomaterials-2082030-supplementary.pdf]

Supporting Information

Improving the Stability of Halide Perovskite Solar Cells Using Nanoparticles of Tungsten Disulfide

Philip Nathaniel Immanuel [1,2,†], Song-Jeng Huang [1,†], Viktor Danchuk [2], Anastasiya Sedova [2], Johnathan Prilusky [2], Achiad Goldreich [2], Hila Shalom [2], Albina Musin [3] and Lena Yadgarov [2,*]

[1] Department of Mechanical Engineering, National Taiwan University of Science and Technology,
Taipei 106, Taiwan; nathaniel.philip@gmail.com (P.N.I.); sgjghuang@mail.ntust.edu.tw (S.-J.H.)
[2] Department of Chemical Engineering, Faculty of Engineering, Ariel University, Ariel 4076414, Israel; viktorde@ariel.ac.il (V.D.); nastiona212@gmail.com (A.S.); jonathanprilusky@gmail.com (J.P.); achiad132@gmail.com (A.G.); hila.shalom1@msmail.ariel.ac.il (H.S.)
[3] Physics Department, Faculty of Natural Sciences, Ariel University, Ariel 4076414, Israel; albinam@ariel.ac.il
* Correspondence: lenay@ariel.ac.il
† These authors contributed equally to this work.

Abbreviations

$MAPbI_3$ - methylammonium lead iodide

WS_2 NPs- Tungsten disulfide Nano particles

C1 – 1 wt% concentration of WS_2 NPs,

C2 – 2 wt% concentration of WS_2 NPs,

C3 – 3 wt% concentration of WS_2 NPs.

SEM – Scanning Electron Microscope

XRD- X-ray Diffraction

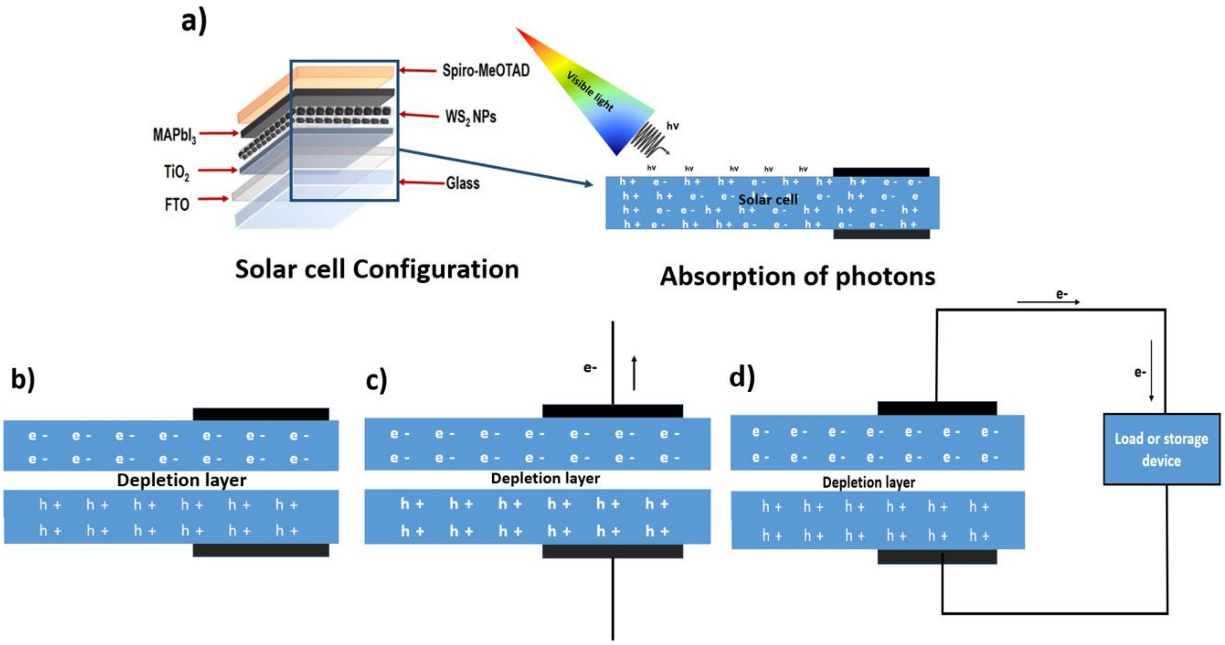

Scheme S1. a) absorption of photons with WS_2 NPs/$MAPbI_3$, b) creation of free charge carriers: separation of electron-hole pair by internal field, c) collection and d) transportation of photo-generated charge carriers through an electrical circuit.

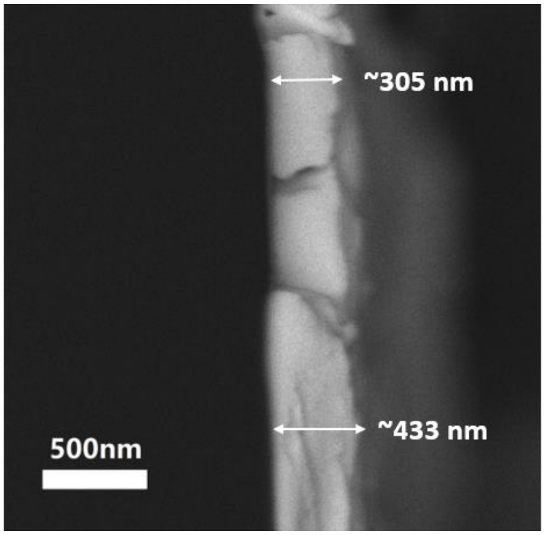

Figure S1. Cross section SEM images of $MAPbI_3$ layer.

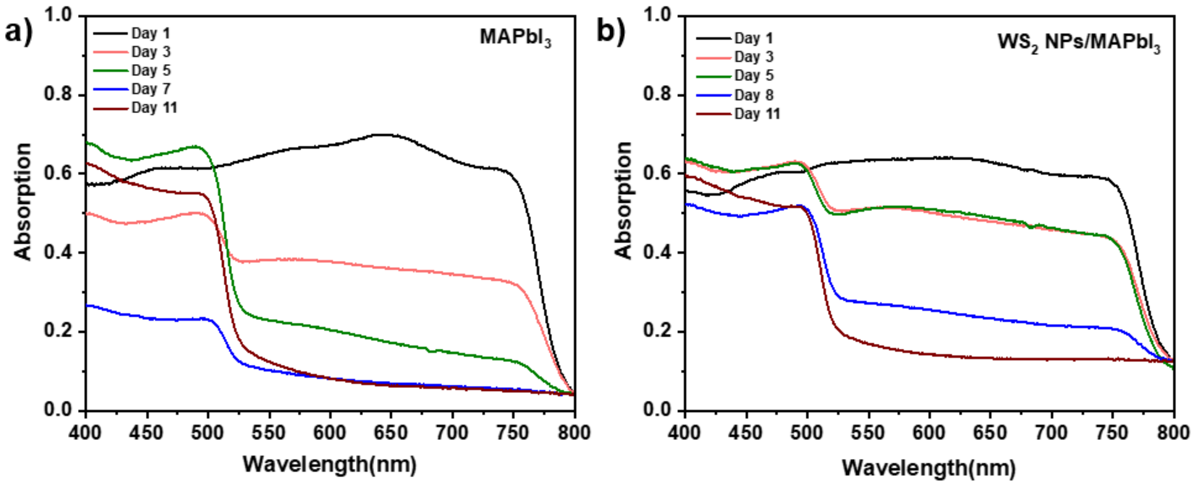

Figure S2. Stability measurement of a) $MAPbI_3$ and b) WS_2 NPs/$MAPbI_3$ films in petri dish.

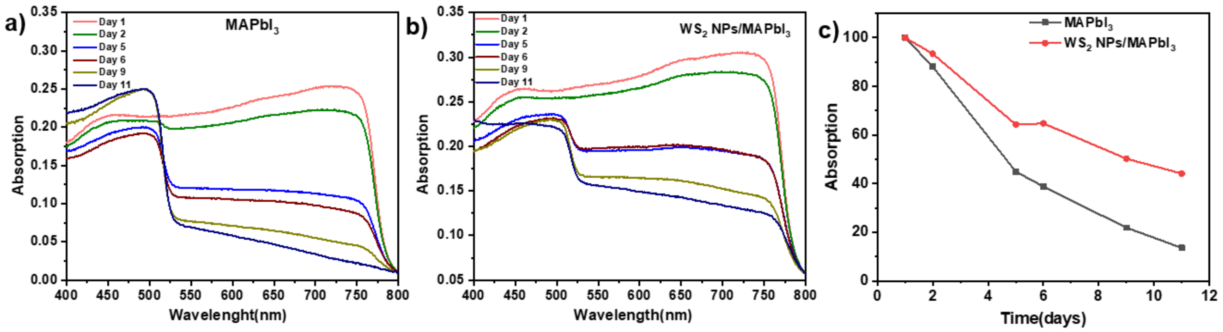

Figure S3. Stability measurement of a) $MAPbI_3$, b) WS_2 NPs/$MAPbI_3$ films on vacuum sealed bags, c) absorption percentage of the hybrid WS_2 NPs/$MAPbI_3$ (red line) and the pristine $MAPbI_3$ (black line).

In addition to the stability test described in the main test, we examined the stability of the $MAPbI_3$ and WS_2 NPs/$MAPbI_3$ film in vacuum sealed bag under sunlight **(Figure S4).** Compared with the previous results **(Figure 4c, main text)**, the WS_2 NPs/$MAPbI_3$ absorption dropped faster. One of the possible reasons for this inconsistency is the amount of the absorbed light (radiation) and heat. Namely, when the samples were placed in a petri dish, the light was absorbed not only by the examined films and the covering bag, but also by the petri dish. Thus, the WS_2 NPs/$MAPbI_3$ and $MAPbI_3$ were exposed to sunlight less than the films examined on the vacuum-sealed bags. Nevertheless, at eleventh days, the absorption rate of the hybrid was ~45% whereas the absorption of $MAPbI_3$ film was only 10%.

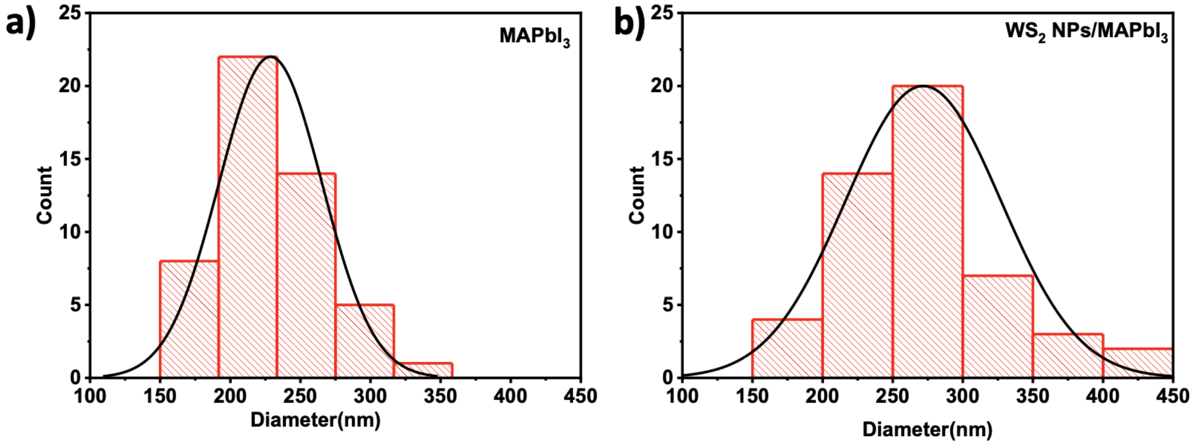

Figure S4. Grain size distribution of a) $MAPbI_3$ and b) WS_2 NPs/$MAPbI_3$ with 1 wt%.

(The analysis is done on SEM micrographs of **Figure 2a and b** in the main text)

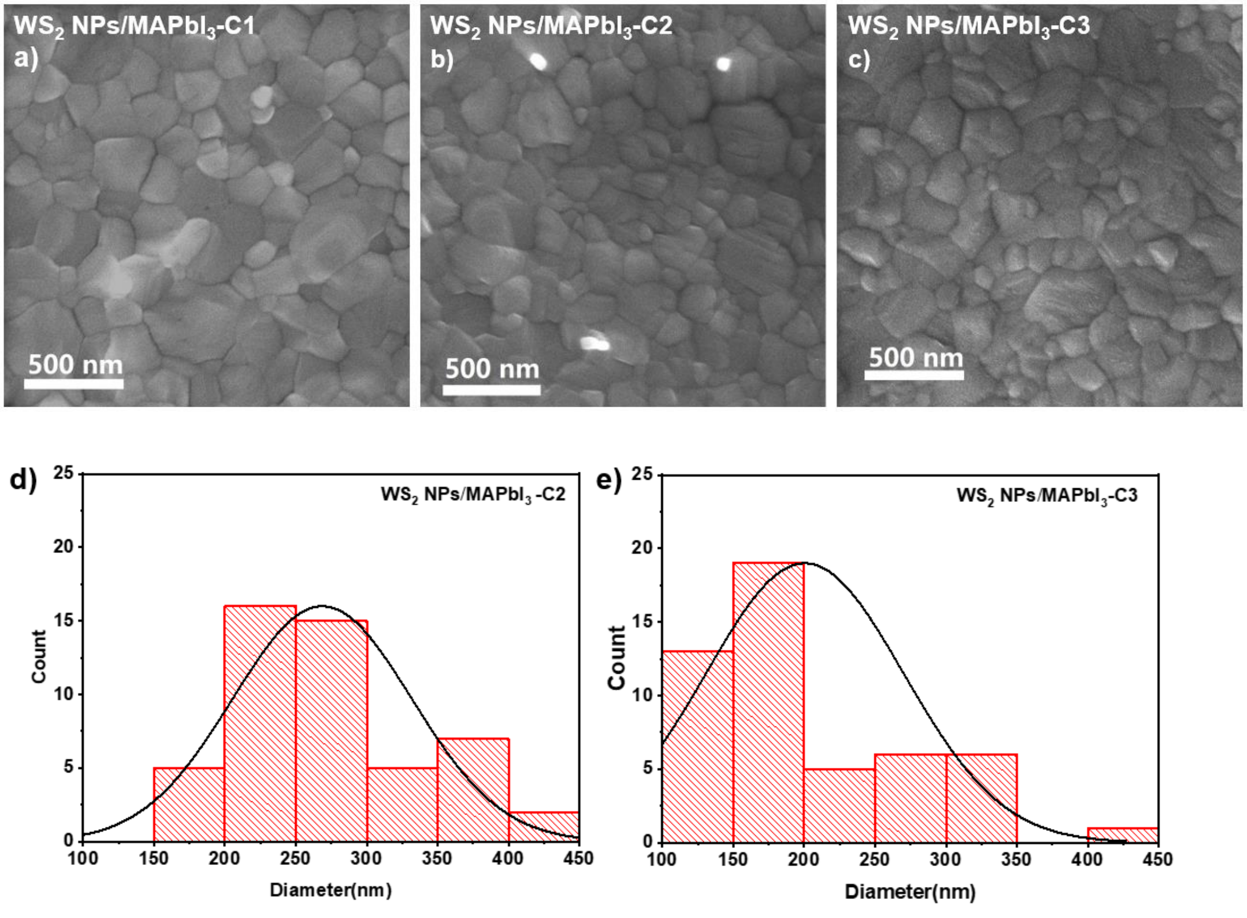

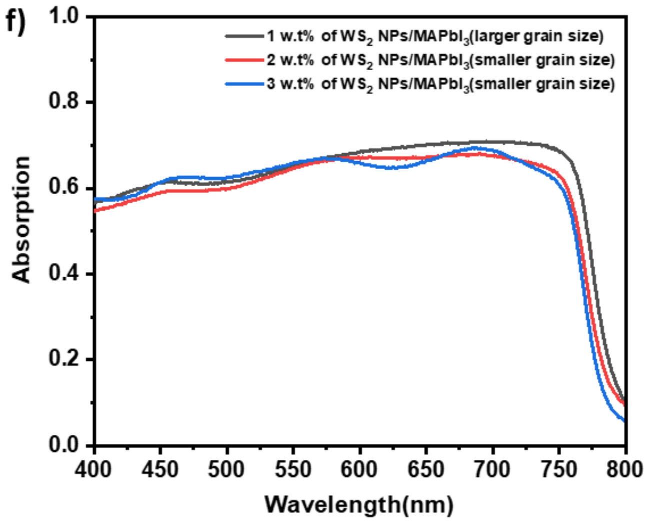

Figure S5. (a-c) SEM images of the WS_2 NPs/$MAPbI_3$ layers with increased concentration of WS_2 NPs. Here C1 – 1 wt% concentration of WS_2 NPs, C2 – 2 wt% concentration of WS_2 NPs, C3 – 3 wt% concentration of WS_2 NPs. d) Grain size distribution of a) WS_2 NPs/$MAPbI_3$ for C2 concentration of NPs and e) WS_2 NPs/$MAPbI_3$ for C3 concentration of NPs. f) Absorbance spectrum of $MAPbI_3$ with three grain size.

The enhancement in the absorption of $MAPbI_3$ film due to the high absorption coefficient of WS_2 NPs. On the other hand, the larger grain size of $MAPbI_3$ is due to the distribution of WS_2 NPs on the substrate. When the concentration of the WS_2 NPs is higher (2 w.t% or 3 w.t%), more aggregation occurs, which leads to uneven size distribution in grains. Although the absorption enhanced, this is not due to the grain size. The absorbance spectrum of $MAPbI_3$ for three-grain size was shown in **Figure S5 (f).**

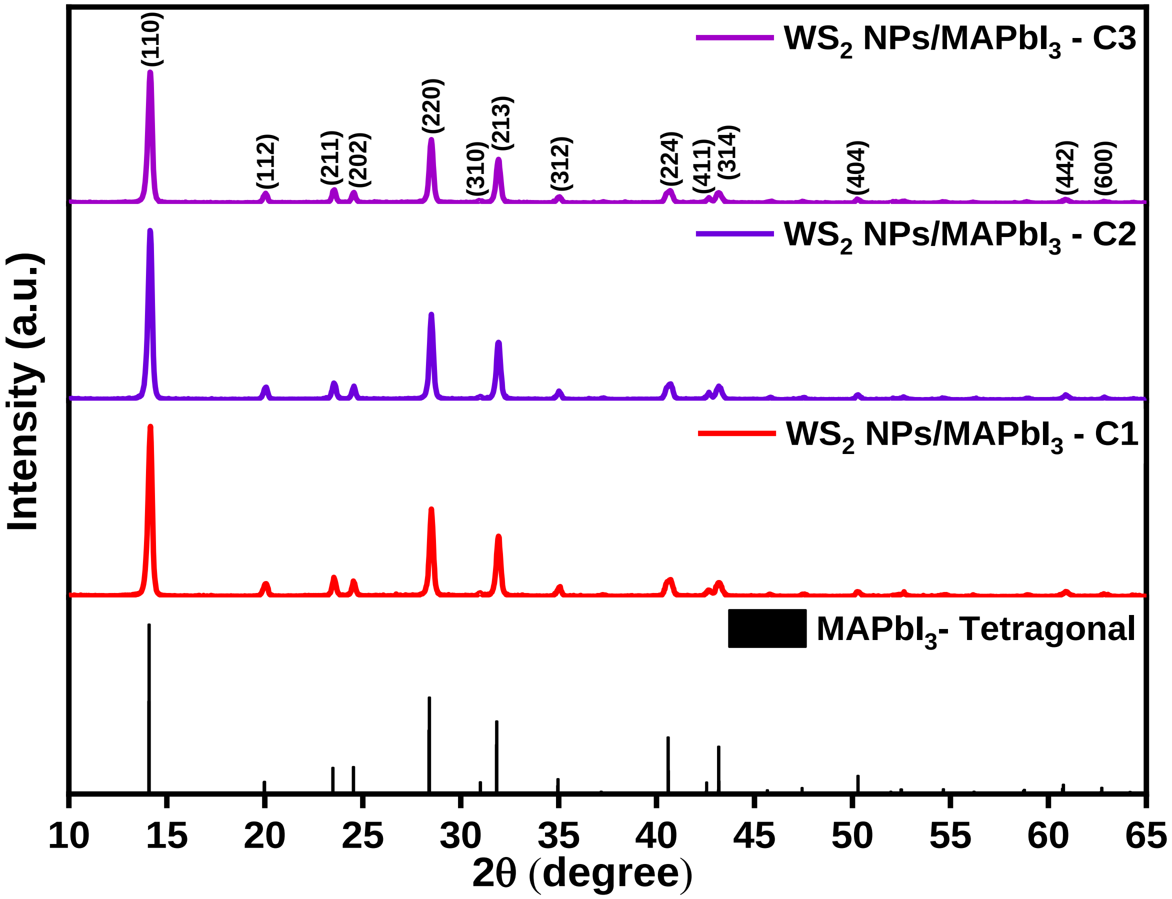

Figure S6. XRD patterns of the WS_2 NPs/$MAPbI_3$ layers with increased concentration of WS_2 NPs. Here C1 - 1 wt% concentration of WS_2 NPs, C2 - 2 wt% concentration of WS_2 NPs, C3 - 3 wt% concentration of WS_2

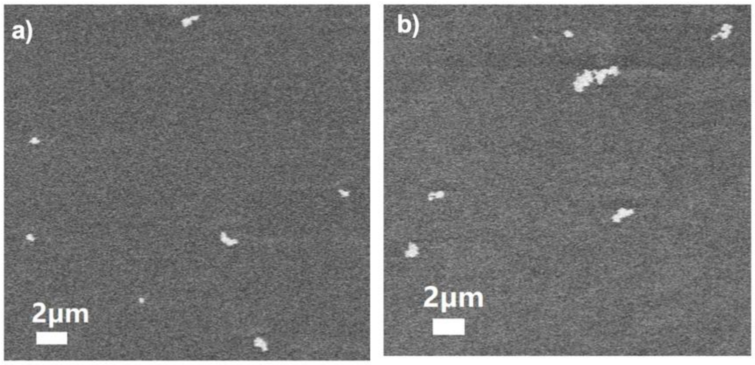

Figure S7. a) SEM images of WS_2 NPs on glasses with C1 concentration and b) SEM images of WS_2 NPs on glasses with C3 concentration.

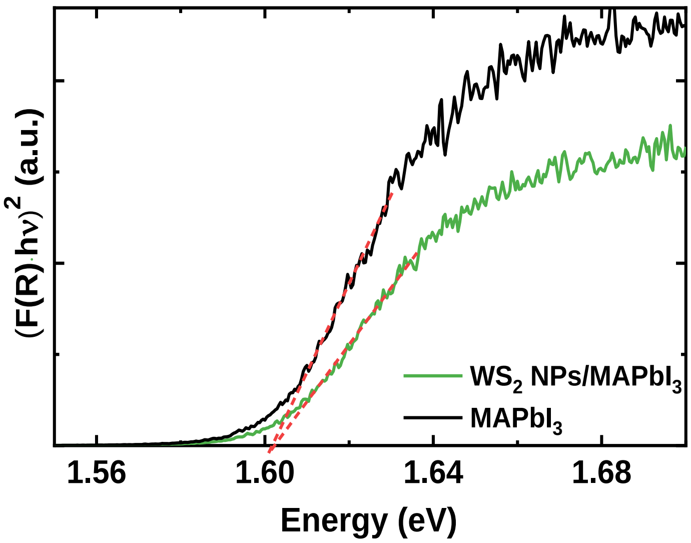

Figure S8. Calculated band gap of $MAPbI_3$ and WS_2 NPs/$MAPbI_3$

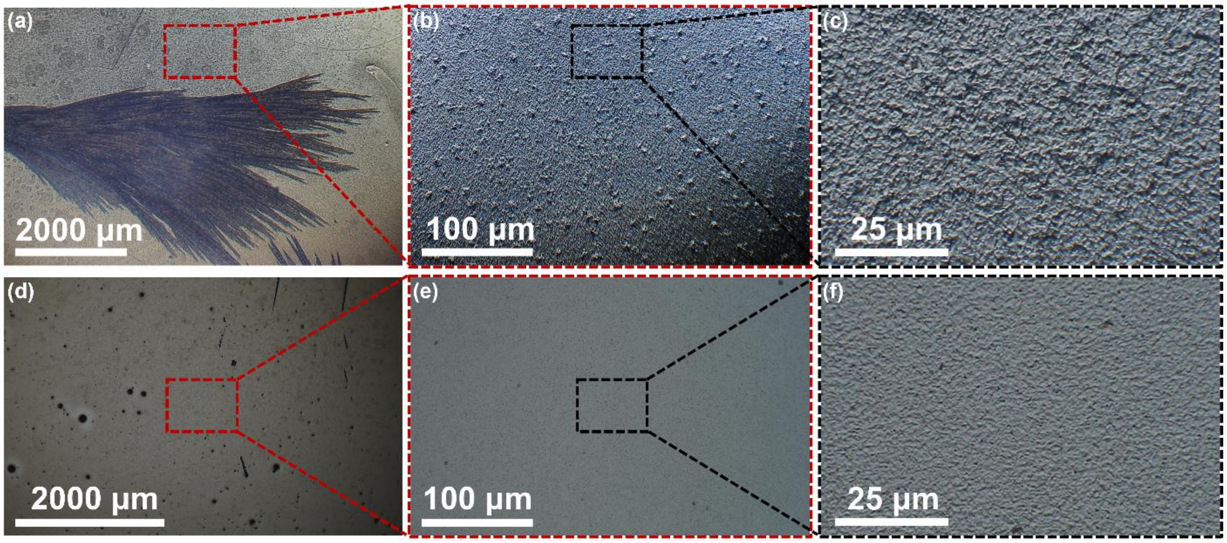

Figure S9. Light microscope images of three different magnifications of the (a-c) $MAPbI_3$ and (d-f) WS_2 NPs/$MAPbI_3$ layers on the FTO glass after exposure to air.

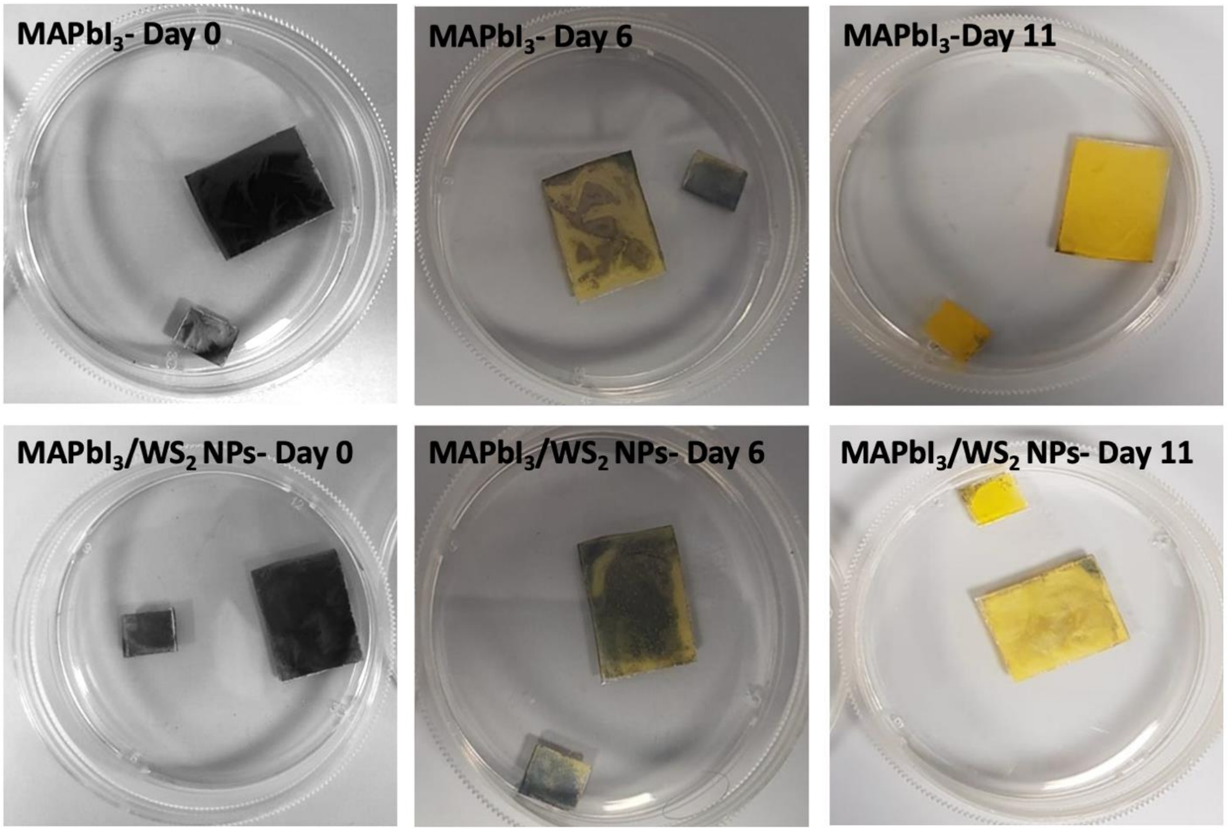

Figure S10. Picture of $MAPbI_3$ films and WS_2 NPs/$MAPbI_3$ films after stability test under sun light.
